# Does vestibular rehabilitation improve postural control of subjects with chronic subjective dizziness?

**DOI:** 10.1371/journal.pone.0238436

**Published:** 2020-09-02

**Authors:** Mine Baydan, Oznur Yigit, Songul Aksoy

**Affiliations:** 1 Department of Audiology, Ankara University, Ankara, Turkey; 2 Department of Audiology, Hacettepe University, Ankara, Turkey; University of Rochester, UNITED STATES

## Abstract

Postural control is the ability to maintain equilibrium and orientation in a gravitational environment. Patients with chronic subjective dizziness have some troubles with their postural stability. The present study aimed to assess the benefit of home-based vestibular rehabilitation in patients with chronic subjective dizziness using computerized dynamic posturography. Therefore, 100 subjects, between 19 to 86 years, diagnosed with dizziness were included in the study. Computerized dynamic posturography was performed to assess postural stability. Vestibular rehabilitation programs included exercises tailored to the particular needs of each patient. After vestibular rehabilitation, patients were re-examined using the same tests. Posturographic data were analyzed and compared for before and after vestibular rehabilitation findings. The mean composite scores before the intervention (58,92 ±11,64) was significantly (p<0.01) lower than the mean composite scores after vestibular rehabilitation (73,83 ± 8,26). This result is found to be statistically significant. In conclusion it could be suggested that the effectiveness of vestibular rehabilitation could be verified by means of computerized dynamic posturography as a concrete method.

## 1. Introduction

Dizziness, is an umbrella term which is used to define a subjective sensation of disorientation or involuntary motion that could be related to several factors [1,2]. Chronic subjective dizziness is defined as follows: persistent sensation of dizziness with subjective imbalance, chronic hypersensitivity to movement without any physical neurotologic illness, medical condition or medication causing dizziness [2,3].

Vestibular rehabilitation (VR) has become widely used in the management of patients with dizziness, disequilibrium, and gait instability [4]. VR is a treatment option for the management of vertigo and unsteadiness, which may cause recovery or improvement of symptoms [5].

Vestibular rehabilitation attempts to improve the competence and well being of the individual who has a vestibular pathology in the performance of daily life activities. It also aims to restore spatial orientation as much as possible. It stimulates visual stabilization, reduces discomfort during head movements, and leads to more excellent stability in body posture while both moving and relaxing [1].

Computerized Dynamic Posturography (CDP) is a test used to evaluate the general assessment of balance, combining labyrinthine, visual and somatosensory data. The most commonly used test is the Sensory Organization Test [6], consisting of six sensory conditions using of a moving platform (somatosensory data), the presence or absence of vision (visual data) and vestibular calibration [7]. Posturography demonstrates which sensory systems are used to maintain balance and the functional use of the vestibular system to provide postural control [8].

The study aims to determine whether home-based vestibular rehabilitation improves postural control of patients with chronic subjective dizziness with computerized dynamic posturography.

## 2. Material and method

The study was approved by Hacettepe University Faculty of Medicine Non-Interventional Clinical Research Ethics Committee (No:GO 19/27, 2019). According to the national legislations and institutional rules and procedures, all patients sign an informed-consent approving analysis of their clinical records, and publication of the anonymous data.

### 2.1.Subjects

Subjects were identified through a retrospective medical record review of patients seen for vestibular and balance rehabilitation between January 2010 and November 2018 at the Balance Rehabilitation Program of the Hacettepe University Audiology Clinic. A hundred subjects diagnosed with chronic subjective dizziness were included in the present study. Inclusion and exclusion criteria were defined as:

Inclusion Criteria: Individuals of both genders over 18 years old who presented with chronic vestibular dysfunction, dizziness or balance impairment and completed the vestibular rehabilitation program.

Exclusion Criteria: Individuals with severe visual and/or hearing impairment, orthopedic disorders that limited the performance of proposed activities, nervous system injuries that resulted in additional motor and/or sensitive damages, and/or peripheral vestibular diseases of the benign paroxysmal positional vertigo or Meniere disease.

### 2.2. Evaluation 1: Computerized dynamic posturography

On the initial visit, the physical therapist / audiologist performed a complete medical, symptom, and functional history, and a physical evaluation. The physical evaluation included a neuromuscular assessment, oculomotor and VOR testing such as saccadic and pursuit eye movements, optokinetic, gaze and spontaneous nystagmus, head impulse test, positional and movement testing, balance testing, and gait evaluation. Next, the individuals with dizziness were subjected to CDP with Equitest SystemTM—Version 4.0 equipment, produced by NeuroCom InternationalTM- USA. SOT, one to CDP's subtest was performed. A harness was provided for safety. The step width during standing was standardized according to the subjects' height.

The conventional SOT is a test that consists of 6 different conditions used to identify relative contribution of the three main sensory systems involved in balance: namely somatosensory, visual and vestibular systems. The 6 conditions include are as follows: (1) eyes open with fixed surface and visual surround, (2) eyes closed with a fixed surface, (3) eyes open with fixed surface and sway referenced visual surround, (5) eyes closed with a sway referenced surface, and (6) eyes open with sway referenced surface and visual surround. By controlling the usefulness of the sensory (visual and proprioceptive) information through sway referencing and/or eyes-open/closed conditions, the SOT protocol systematically eliminates useful visual and/or support surface information and creates sensory conflict situations [7–10].

Three trials were taken for each condition. A fall was defined as a patient taking a step, grabbing the wall or side the platform, opening their eyes during an eyes closed condition, or when the patient exceeded their limits of stability. If the operator stopped the trial, the trial was scored a 0.

Based on the data under each condition, Equitest is capable of calculating the Average for each of them, an index for somatosensory, visual and vestibular functions and the relationship between visual and vestibular information, known as "visual preference," in addition to a balance index, all of which are described below:

Somatosensory function (SOM): Average of condition 2 / Average of condition 1

Visual function [8]: Average of condition 4 / Average of condition 1

Vestibular function (VEST): Average of condition 5 / Average of condition 1

Visual preference: Average of condition 3+6 /Average of condition 2+5 [7].

The "Equilibrium Score" is based on the assumption that a normal person can sway 12.5 degrees without losing balance within the limits of stability [11]. When calculating the balance score, the subject’s maximum anterior-posterior gravitational center ossicilations are compared the maximum theoretical limits [12]. The composite equilibrium score was recorded for analysis that was automatically computed by the algorithm software. The score can range from 0 to 100 indicating no movement on the posture platform. Higher SOT scores indicate greater stability [13]. The results of the posturographic assessment are compared to age-matched norms.

### 2.3. Intervention: Vestibular rehabilitation

Once the first posturography session had been performed for each of the individuals to be studied, the home-based vestibular rehabilitation (VR) program was begun. A customized exercise program was developed for each subject according to the results of the assessment and included the following interventions as indicated: gaze stabilization, balance and gait training, and habituation exercises. The vestibular rehabilitation program was tailored as follow: (a) if just the VEST parameter was under the normal limits vestibulo-ocular reflex (VOR) exercises, (b) if the VEST and SOM parameters were under the normal limits together VOR exercises on foam-pad and (c) if the VIS and VEST parameters were under the normal limits VOR and foam pad exercises have been suggested (Table 1). Although the planning of the exercise program varies according to the condition of each patient, the exercises are planned to perform twice a day.

**Table 1:** Vestibular rehabilitation program.

Rehabilitation Group	Duration of Rehabilitation	Type of Exercise	Dosage of Exercise
VEST parameter under normal limits	4	Cawthorne-Cooksey Exercises	
	Week 1–2	Gaze Stabilization Exercises in Sitting Position	Twice a day for 5–8 minutes
	Week 3	Gaze Stabilization Exercises in Standing Position	Twice a day for 5–8 minutes
	Week 4	Gaze Stabilization Exercises While Moving Around	Twice a day for 5–10 minutes
VEST-SOM parameters under normal limits	4		
	Week 1	Standing on foam pad (Eyes open)	Twice a day for 3–5 minutes
	Week 2	Standing on foam pad (Eyes closed)	Twice a day for 5–8 minutes
	Week 3–4	Gaze Stabilization Exercises on Foam pad	Twice a day for 5–8 minutes
VIS-VEST Parameters under normal limits	6		
	Week 1–2	Gaze Stabilization Exercises in Sitting Position	Twice a day for 5–8 minutes
	Week 3	Gaze Stabilization Exercises in Standing Position	Twice a day for 5–8 minutes
	Week 4	Gaze Stabilization Exercises While Moving Around	Twice a day for 5–10 minutes
	Week 5–6	Gaze Stabilization Exercises on Foam pad	Twice a day for 5–8 minutes

### 2.4. Evaluation 2: Computerized dynamic posturography

At the end of the 4–6 weeks of vestibular rehabilitation, patients were reassessed using computerized dynamic posturography.

### 2.5. Statistical analysis

Data were analyzed using SPSS 21.0 (Statistical Package for Social Sciences) program. A p-value below 0.05 was considered statistically significant. Descriptive analysis (mean and standard deviation) included demographic factors such as age and sex. A paired t-test was used to compare SOT scores before and after vestibular rehabilitation.

## 3. Results

In total, 100 individuals (63 female and 37 male, 19–86 years) who diagnosed with dizziness were evaluated. The mean age of the subjects was 47.24 ± 15.57 years.

**Fig 1:**
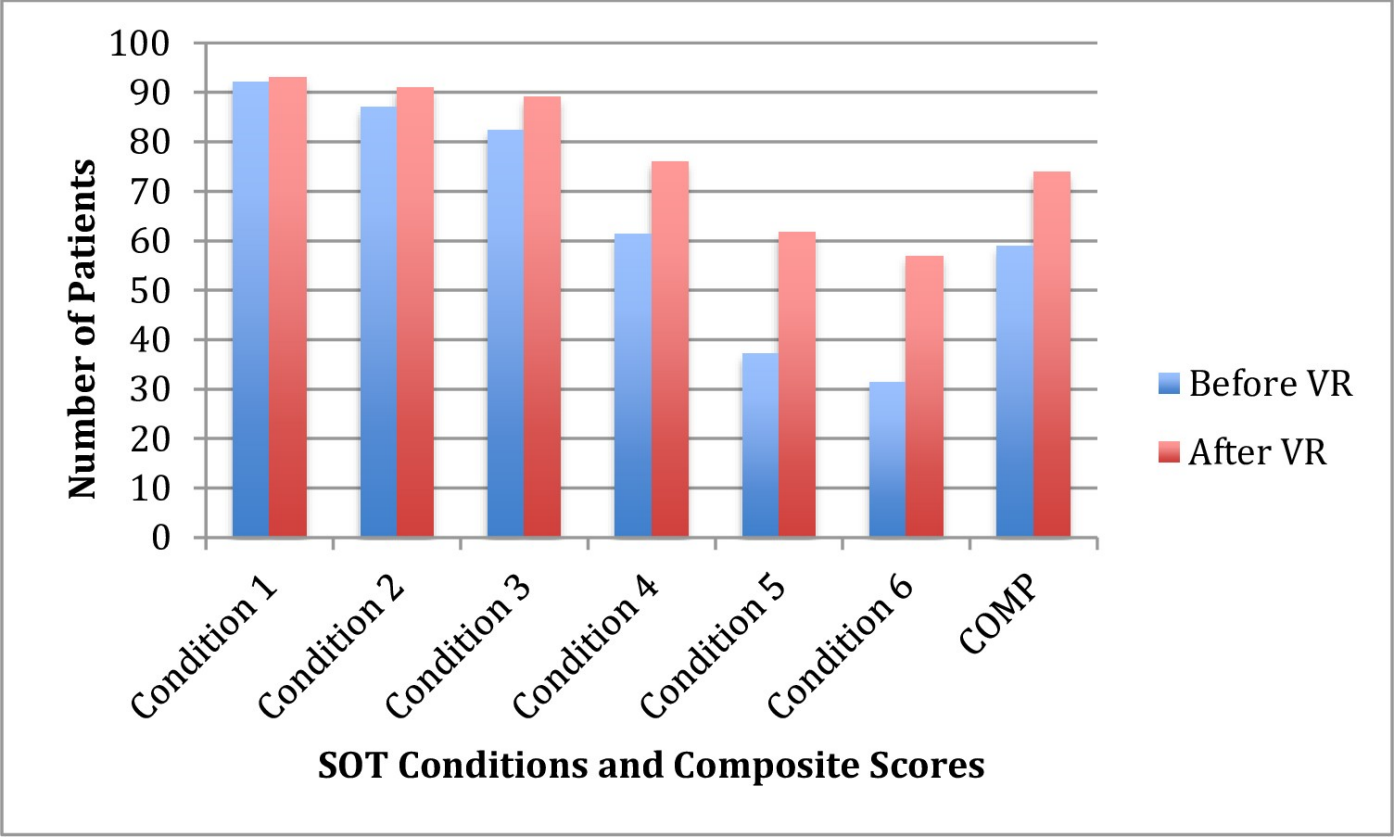
SOT conditions improvements before and vestibular rehabilitation.

The comparison of average results under each of the various computerized dynamic posturography conditions studied, before and after vestibular rehabilitation, can be found in Table 2 and Fig 1. We observed a significant increase in the numerical values produced under all conditions. Substantially, there was a significant improvement in Condition 5, which is the best condition to evaluate the vestibular system, since visual and proprioceptive information is excluded. We also observed a significant increment in composite scores after vestibular rehabilitation.

**Table 2:** Means several SOT conditions before and vestibular rehabilitation.

Equilibrium Score	Mean Before VR	Mean After VR	p
Condition 1	92.13 ± 4.44	93.09 ± 4.96	0,019
Condition 2	87.07 ± 9.62	91.02 ± 5.31	0,0001
Condition 3	82.41 ± 14.40	89,14 ± 7.18	0,0001
Condition 4	61.31 ± 17.05	76.06 ± 11.62	0,0001
Condition 5	37.22 ± 17.87	61.74 ± 11.78	0,0001
Condition 6	31.47 ± 20.26	56.94 ± 15.81	0,0001
COMP	58.92 ± 11.64	73.83 ± 8.27	0,0001

The number of patients who have normal and abnormal results before and after vestibular rehabilitation in SOM, VIS, VEST and PREF parameters of SOT was given in Table 3. As a result, it can be said that CDP can be used both determine the disoriented system and the effectiveness of vestibular rehabilitation.

**Table 3:** Number of subjects with normal and abnormal SOT parameters before and after vestibular rehabilitation.

		Pre-VR (Number of Subjects)	Post-VR (Number of Subjects)
**SOM**	Normal	12	100
	Abnormal	88	0
**VIS**	Normal	36	84
	Abnormal	64	16
**VEST**	Normal	24	88
	Abnormal	76	12
**PREF**	Normal	62	98
	Abnormal	38	2

## 4. Discussion

The primary purpose of the present study was to investigate whether the computerized dynamic posturography is beneficial for assessing the effectiveness of vestibular rehabilitation in subjects with chronic subjective dizziness. Our results revealed that the performing computerized dynamic posturography before and after vestibular rehabilitation is useful to determine the effectiveness of vestibular rehabilitation.

The main problem encountered when dealing with subjective dizziness and/or disequilibrium is the difficulty to measure symptoms objectively. Distinguishing normal and abnormal balance function is an important component of the diagnostic investigation of all patients with dizziness and balance complaints. Posturography has yielded results on sway on the sensory organization test that have been associated with a variety of balance disorders [14,15]. Posturography is a method of measuring the proportion in which different inputs participate at maintaining the equilibrium: the visual input, the vestibular input and the proprioceptive input [16]. These data provide crucial information in determining to vestibular rehabilitation approach. To give an instance, if the visual parameter [17] is under the normal limits vestibular rehabilitation can be based on the vestibulo-ocular reflex (VOR) exercises or if the somatosensory and vestibular parameter is under the normal limits vestibular rehabilitation can be based on VOR and vestibulo-spinal reflex (VSR) exercises.

A customized vestibular rehabilitation therapy program is an important treatment modality for patients who have dizziness, disequilibrium and gait disturbances [1]. After determining an appropriate vestibular rehabilitation approach, another critical issue is to assess the effectiveness of this rehabilitation. It is crucial to assess the effectiveness of vestibular rehabilitation because it is necessary to change the approach or recreate a vestibular rehabilitation program if the intervention does not benefit.

Most clinical studies on the effectiveness of vestibular rehabilitation have relied on subjective measures such as questionnaires and scales focused on assessing changes in symptom severity [4,5,7,8,13,17,18]. Besides these studies, many other recent studies have been used objective measures for assessing the effectiveness of vestibular rehabilitation [19–25]. Aforementioned objective measures are Computerized Dynamic Posturography [19,21–25], Dynamic Visual Acuity Test [19,24], Video-oculoscopy [19] and Functional Reach Test [20]. Compared to other objective measures, the advantages of computerized dynamic posturography are: evaluating the up-right balance and thus a making a functional measurement due to vestibulo-spinal function, providing information other than those obtained from vestibulo-ocular tests such as caloric and rotational tests and is easily manageable, repeatable and fast. Also, the abnormalities are usually nonspecific and nonlocalizing and do not provide information about the activity of specific labyrinthine sensors or the laterality or etiology of any disturbance. Despite these disadvantages; computerized dynamic posturography is an appropriate test for patients with symptoms of dizziness or when accompanied by symptoms of imbalance when walking [26].

In the present study, CDP used to determine the effectiveness of vestibular rehabilitation. For this purpose, subjects were administered SOT before vestibular rehabilitation to specify which system or systems used to maintain balance was disoriented and then the vestibular rehabilitation program was tailored based on this data. After the vestibular rehabilitation program, reassessment was performed again with SOT to check if there is an improvement or not. The strong correlation was found in equilibrium scores for all conditions and composite scores before and after vestibular rehabilitation.

Medeiros et al. (2003), has found a significant improvement condition 1 and condition 5 of the equilibrium scores and of the composite balance score in ten children with vestibular symptoms [21]. Cass et al. (1996) compared the SOT results before and after vestibular rehabilitation in 67 patients with complaints of vertigo, dizziness or imbalance. The results indicate that 60% of patients showed objective improvement of balance function; 25% of patients improved to normal function [22]. Black et al. (2000) examined the 18 patients with chronic vestibular complaints and chief complaints of unsteadiness, imbalance and/or motion intolerance before and after rehabilitation using SOT. Subjects showed statistically significant improvements in SOTs and overall composite score [23]. Morisod et al. (2017) reported that 24 of 42 patients (57%) with chronic subjective dizziness showed an improvement or normalization on their CDP [25]. In this respect, the findings we obtained are similar to those in the literature. Clinical improvement is easily distinguished by the therapist, otorhinolaryngologists and subject’s himself/herself [21].

Since none of the individuals with a diagnosis of CSD presented with an identifiable ontological contribution, we speculated that observed decreades in level of dizziness could be explained by vestibular rehabilitation. However, such improvement could be explained by the reduced anxiety experienced by the patients [2]. This fact could be a limitation for our study. Future researches could be planned investigate such relations.

In this study statistical analysis implemented on SOT showed that the significant improvement in the composite score is primarily due to improvements under condition 5 and 6. The improvement in sensory analysis of final vestibular function was also significant, further indicating objective and effective improvement under condition 5, which performed with an unstable platform and with closed eyes, revealing the subject’s use of the vestibular information in maintaining an upright position. This finding is compatible with other studies in the literature [21,25,27].

## Conclusion

It is crucial to determine which system is disoriented in maintaining balance to provide beneficial vestibular rehabilitation. Moreover, it is also essential to assess the effectiveness of vestibular rehabilitation. It could be speculated that Computerized Dynamic Posturography could be a beneficial method to determine the disoriented system and the effectiveness of vestibular rehabilitation. It could provide objective data to compare the results of a vestibular rehabilitation.

## Supporting information

S1 Data (XLSX)
